# Hormone suppression silencing GUCY2C is required for colorectal cancer

**DOI:** 10.1186/2050-6511-16-S1-A31

**Published:** 2015-09-02

**Authors:** Scott A Waldman

**Affiliations:** 1 Department of Pharmacology and Experimental Therapeutics, Thomas Jefferson University, Philadelphia, PA 19107, USA

## 

Colorectal cancer is the fourth most common cancer and the second leading cause of cancer death. Transformation begins with activation of Wnt signaling through mutations in APC (80%) or its degradation target β-catenin (15%), producing a gain-of-function in TCF-dependent nuclear transcription underlying epithelial dysfunction and tumorigenesis. While a role for APC and β-catenin in colorectal cancer is well-established, steps leading from gene mutation to tumorigenesis, and their reversibility, remain incompletely defined. Guanylin is the paracrine hormone in colorectum for the receptor GUCY2C. This hormone is the most commonly lost gene product in colorectal cancer, and its universal suppression and associated silencing of GUCY2C at the earliest step in neoplasia contributes to tumorigenesis in a mechanism that is conserved across species. GUCY2C regulates homeostatic mechanisms organizing the intestinal crypt-surface axis, and its silencing through guanylin suppression drives hyperproliferation, DNA damage, metabolic reprogramming and desmoplasia contributing to tumorigenesis. Beyond this dysregulation of homeostatic mechanisms contributing to transformation, we recently discovered that guanylin suppression is required for tumorigenesis induced by mutant APC-β-catenin, reflecting a role for GUCY2C in β-catenin degradation that blocks tumorigenesis. Thus, inactivation of APC, or activation of β-catenin, induces TCF-dependent elimination of guanylin transcription and translation in human intestinal cells in vitro and in conditional genetic mouse models in vivo. Conversely, activation of GUCY2C induces cGMP protein kinase-dependent elimination of wild type or mutant β-catenin by amplifying proteosomal degradation, even in the context of mutations which inactivate the APC degradation complex. Importantly, enforced genetic expression of guanylin in intestinal epithelial cells completely eliminated tumorigenesis in all mouse models of colorectal cancer examined. These observations reveal a pathophysiologic model in which mutant APC-β-catenin signaling eliminates guanylin expression as an obligatory step in tumorigenesis. In turn, silencing GUCY2C reversibly disrupts epithelial homeostatic processes corrupted in tumorigenesis, and removes an essential block to transformation, creating a circuit which amplifies mutant APC-β-catenin signaling. These studies shift the prevailing paradigm for colorectal tumorigenesis from an irreversible oncogenomic mechanism to a reversible functional mechanism whose reconstitution abrogates those mutational defects. Indeed, they define a novel molecular pathway that is obligatory for tumorigenesis, to which the transformation process is addicted. In that context, they highlight how this addiction creates a unique disease-specific vulnerability that can be leveraged to eliminate tumorigenesis by GUCY2C hormone replacement. We have translated these observations to an NCI-funded clinical program exploring the utility of oral GUCY2C ligands to prevent colorectal cancer.

